# Supplementary material for: Geophysical constraints on the reliability of solar and wind power worldwide
Source: Nat Commun. 2021 Oct 22;12:6146. doi: 10.1038/s41467-021-26355-z (PMC8536784; doi:10.1038/s41467-021-26355-z)
Supplement: Supplementary file 1 — Supplementary Information [file 41467_2021_26355_MOESM1_ESM.pdf]

Supplementary Information for

Geophysical constraints on the reliability of solar and wind power worldwide

Dan Tong[1,2,3], David J. Farnham[3], Lei Duan[3], Qiang Zhang[1], Nathan S. Lewis[4,3], Ken Caldeira[3,5], and Steven J. Davis[2,3,6]

[1] *Ministry of Education Key Laboratory for Earth System Modeling, Department of Earth System Science, Tsinghua University, Beijing, 100084, China*
[2] *Department of Earth System Science, University of California, Irvine, CA, 92697, USA*
[3] *Carnegie Energy Innovation, Carnegie Institution for Science, Stanford, CA, 94035, USA*
[4] *Division of Chemistry and Chemical Engineering, California Institute of Technology, Pasadena, CA 91125, USA*
[5] *Breakthrough Energy, 4110 Carillon Pt., Kirkland, WA, 98033, USA*
[6] *Department of Civil and Environmental Engineering, University of California, Irvine, CA, 92697, USA*

*corresponding author: dantong@tsinghua.edu.cn

Supplementary Note 1. Explanation for near constant solar resource around daytime peak.

In this work, many of the derived solar capacity factors are quite flat around noon, as opposed to showing a discrete peak near the middle of the day. This midday flatness can be explained by the adjustment of the direct sunlight component as explained below.

During the calculation of solar capacity factors, the incoming surface shortwave radiation is first separated into its direct and diffuse components. The direct solar radiation is then divided by the cosine of the zenith angle to estimate the total incoming direct solar radiation, and multiplied by the incidence angle to estimate in-panel direct radiation. Because the cosine zenith angle is small near noon, it could result in large vertical direction incoming direct radiation. To constrain the abnormally large incoming direct solar radiation, we applied a Beer-Lambert Law using both top-of-atmosphere and surface shortwave radiation, which sets an upper limit for the maximum surface solar radiation. Our analysis shows that due to factors including cloud cover, the Beer-Lambert constraint does not always give a maximum value at the daytime peak hour. The shape of the solar capacity factor daily cycle could be distorted further by averaging across a large geophysical area, in which grid cells at different local times exist. The abovementioned factors together produce the midday plateau in solar capacity curves for many countries presented here.

Supplementary Note 2. The development of statistical model to predict reliability.

In this study, we developed an expression that can be used to predict the reliability given country size, the level of annual generation, and the capacity of energy storage (Supplementary Table 3). The expression (S1) developed here is shown as following:

$$z = \left(1 - \mathrm{e}^{a^{ka} \times \left(\frac{-(\mathrm{b}-1)}{kb} - \frac{s}{ks}\right)}\right)(1 - z_0) + z_0 \quad \text{(S1)}$$

where z represents the predicted reliability with excess annual generation and storage, z_0 represents the reliability with no excess annual generation and no storage, a represents the land area, b represents the level of annual generation relative to annual demand (i.e. 1, 1.5, and 3), s represents the capacity of energy storage relative to mean hourly electricity demand (i.e. hours of storage), ka, kb, and ks represent parameters that are estimated form the Macro Energy Model simulations.

ka, kb, and ks are estimated as 0.149612, 0.419976, and 16.4896, respectively, by means of maximum likelihood. The predicted reliability and actual reliability show a good fit with the correlation coefficient (R) equaling 0.95 (R^2=0.90; Supplementary Figure 15).

Supplementary Note 3. The sensitivity tests of different solar tracking systems and another reanalysis weather data on the electricity system reliabilities.

Our study assumes a horizontal single-axis tracking system when calculating the solar capacity factors. In order to test the impacts of different solar tracking systems on the electricity system reliabilities, we further estimate the solar capacity factors adopting dual-axis (both a horizontal and a vertical axis) tracking system. In our dual-axis assumption, the panels will always be oriented towards the sun with an incident angle of zero, representing the maximum solar-system energy production potential. The reliability changes are shown in Supplementary Figure 10, we find that, the solar tracking systems have small impacts on the electricity system reliabilities and the reliability change ratios are within $\pm$5%. Especially under the high-

level of annual generation relative to annual demand (3x generation; Fig. S10c), the impacts would be very small. it is noted there are relatively large impacts on the system reliabilities in some countries, for example, in Russia, Canada, and Sweden, it is because that these countries are located the high latitude area, and the panels will always be oriented towards the sun with the dual-axis solar tracking system while the tilted panels are used with single-axis tracking system. In summary, there are very small impacts on the system reliabilities regardless of solar tracking systems.

In addition, to investigate uncertainties of our results associated with reanalysis weather data used here (i.e., MERRA-2), we apply the same estimation process for capacity factors of solar and wind using ERA5 provided by European Centre for Medium-Range Weather Forecasts (ECMWF). Hourly historical (from 1980 to 2018) variables, such as top of atmosphere and surface incoming solar radiation, surface air temperature, and 100-meter wind speed that are required to calculate the potential power generation, are downloaded and re-gridded into the same horizontal resolution as MERRA-2. Previous studies comparing MERRA-2 and ERA5 have shown that bias exists in both reanalysis products[30,31]. Our estimates of the system reliabilities by using ERA5 data in the 42 major countries are in good agreement with results of MERRA-2: under 1x generation and the most reliable mixes without storage, reliability under the different loads varies on average from -9.4% to 1.3% (see Fig. S11a). The differences are similar in systems with excess generation (Figs. S11b-c). We also compared the magnitude and duration of unmet demand in 16 major countries like Figure 4 (see Supplementary Figure 12). The data products of MERRA-2 and ERA5 both can essentially capture the number of hours each year that such a gap occurred. By contrast, the MERRA-2 data has a better performance of meeting hourly demand in larger countries (i.e. Russia and Canada) but a similar performance in small countries (i.e. United Kingdom). The somewhat different patterns of resource variability in the two datasets do not alter our main conclusions.

Supplementary Note 4. The sensitivity tests of various demand characteristics.

In this study, only one-year of demand data is employed to assess the geophysical constraints of 39-year solar and wind resources. We realize that load profiles for electricity in many of countries would be different over the past 30 years and in future, there will impact the electricity system reliability, especially under the case of no additional energy storage to dispatch the electricity. Therefore, considering the limits of computing resources, we combined the load profiles of other countries and regions (i.e. 192 countries and regions) and the solar and wind capability (i.e. capacity factors) of the U.S. to evaluate their impacts on the electricity system reliabilities. We analyze systems ranging from 100% solar (no wind) to 100% wind (no solar), in which total annual generation ranged from equal to annual demand ("1x generation") to up to three times of annual demand ("3x generation") with no available energy storage. As shown in Supplementary Figure 14, without any excess annual generation or energy storage, the impacts of various load profiles on the system reliabilities are within $\pm$10% (Fig. S14a), and the most reliable solar-wind generation mixes (25% solar and 75% wind) are not changed. With the level of annual generation relative to annual demand increasing (from "1x generation" to "3x generation"; Fig. S14), the impacts on the electricity system reliabilities are much smaller, especially under the most reliable mixes (i.e. $\leqslant\pm$0.2%). In conclusion, we think there are relatively small impact on reliability especially under the high level of annual generation relative to annual demand, and the impact would be further reduced if there is energy storage.

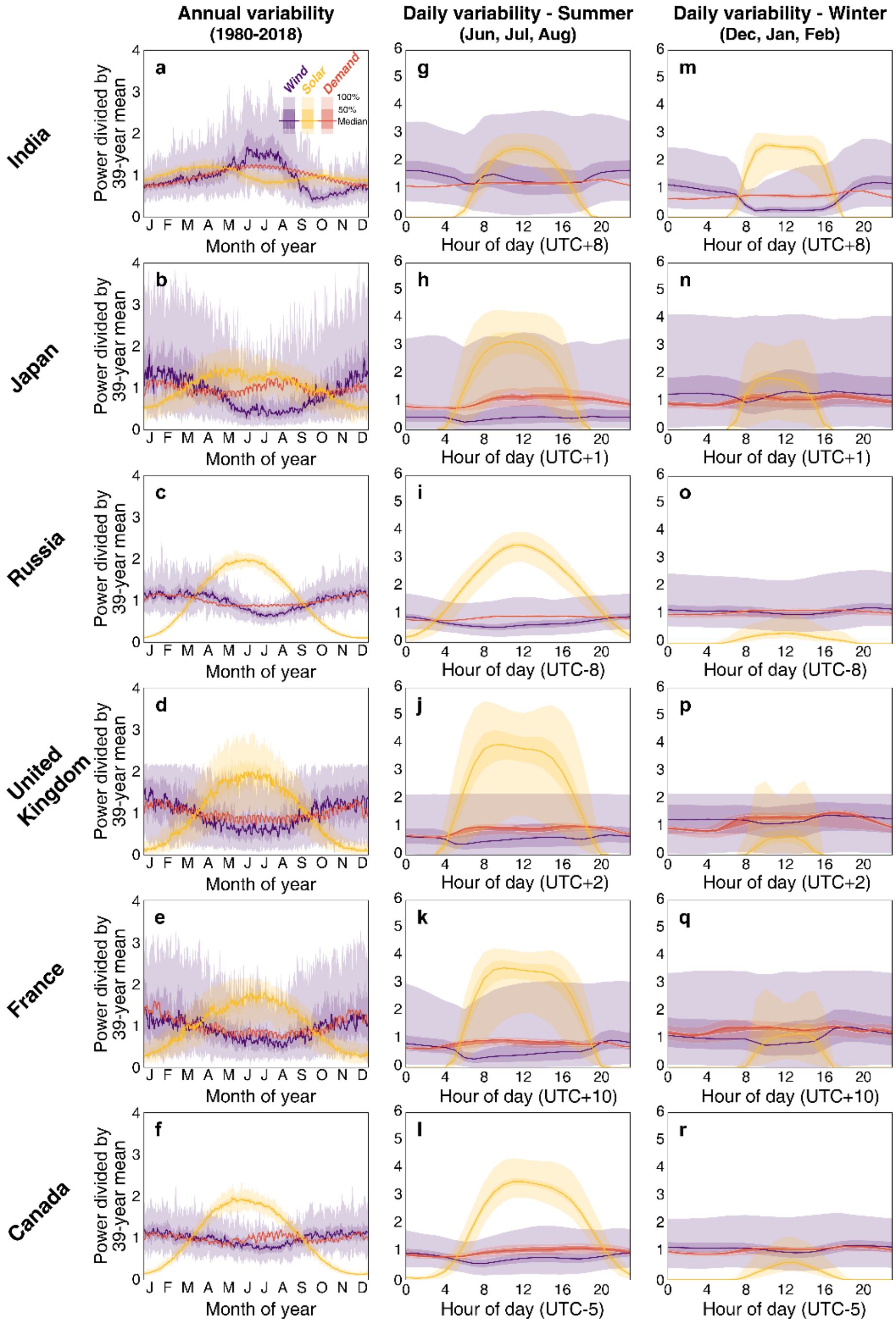

Supplementary Figure 1 | Temporal variability of solar and wind resources and electricity demand. Climatological variability of the area-weighted median power from sun (orange) and wind (blue) resources around each selected country from six continents during the 39-year period 1980-2018: India (a, g, m), Japan (b, h, n), Russia (c, i, o), United Kingdom (d, j, p), France (e, k, q), and Canada (f, l, r). And the left column (a-f) for the daily and seasonal, the middle column (g-l) for hourly summer (June, July, and August), the right column (m-r) for hourly winter (December, January, and February) variability. The lines represent the median, the dark shading represents the inner 50% of observations (25th to 75th percentile) and the light shading represents the outer 50% of observations (0th to 100th percentile). Red curves in each panel represent electricity demand for a single but latest available year for each country. The time of day shown is local time zone of each country and expressed as Coordinated Universal Time (UTC). Note that the middle of local time zones has been selected for the countries with multiple time zones. The solar, wind, and demand data are each normalized by their respective 39-year mean value.

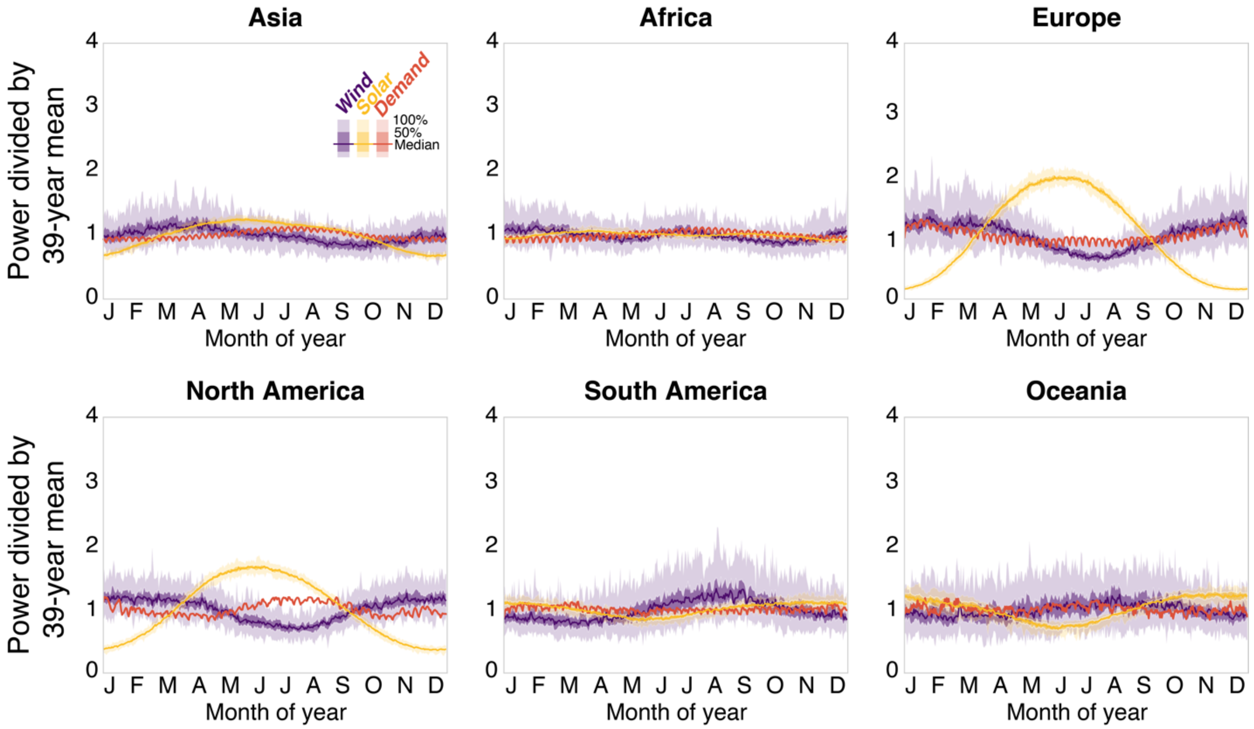

Supplementary Figure 2 | Temporal variability of solar and wind resources and electricity demand.

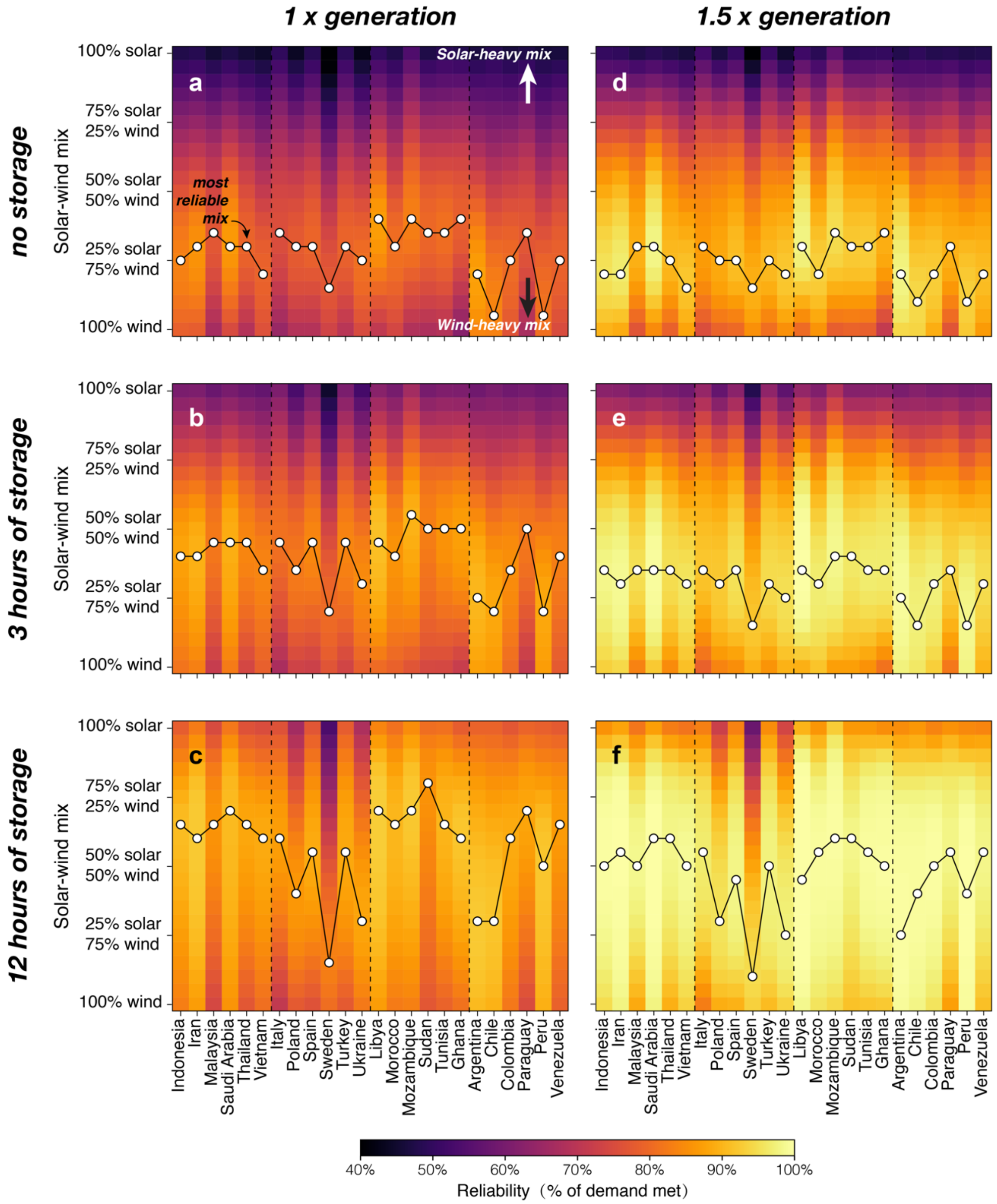

Supplementary Figure 3 | Reliability of electricity supply system by varying the solar and wind resource mix, generation and energy storage. Shading in each panel represent the 39-year average estimated reliability (% of total annual electricity demand met) by a mix of solar and wind resources ranging from 100% solar to 100% wind (every 5% change for solar-wind resource mix). Here 24 main countries are chosen to show their ability to meet total annual electricity, including 24 main countries from four continents (Asia, Europe, Africa, and Americas). The black dots represent the highest reliability within each country under 21 sets of solar and wind mix. Storage and generation quantities are varied in each panel: (a) 1 x generation without storage; (b) 1 x generation with 3 hours of storage; (c) 1 x generation with 12 hours of storage; (d) 1.5 x generation without storage; (e) 1.5 x generation with 3 hours of storage; and (f) 1.5 x generation with 12 hours of storage.

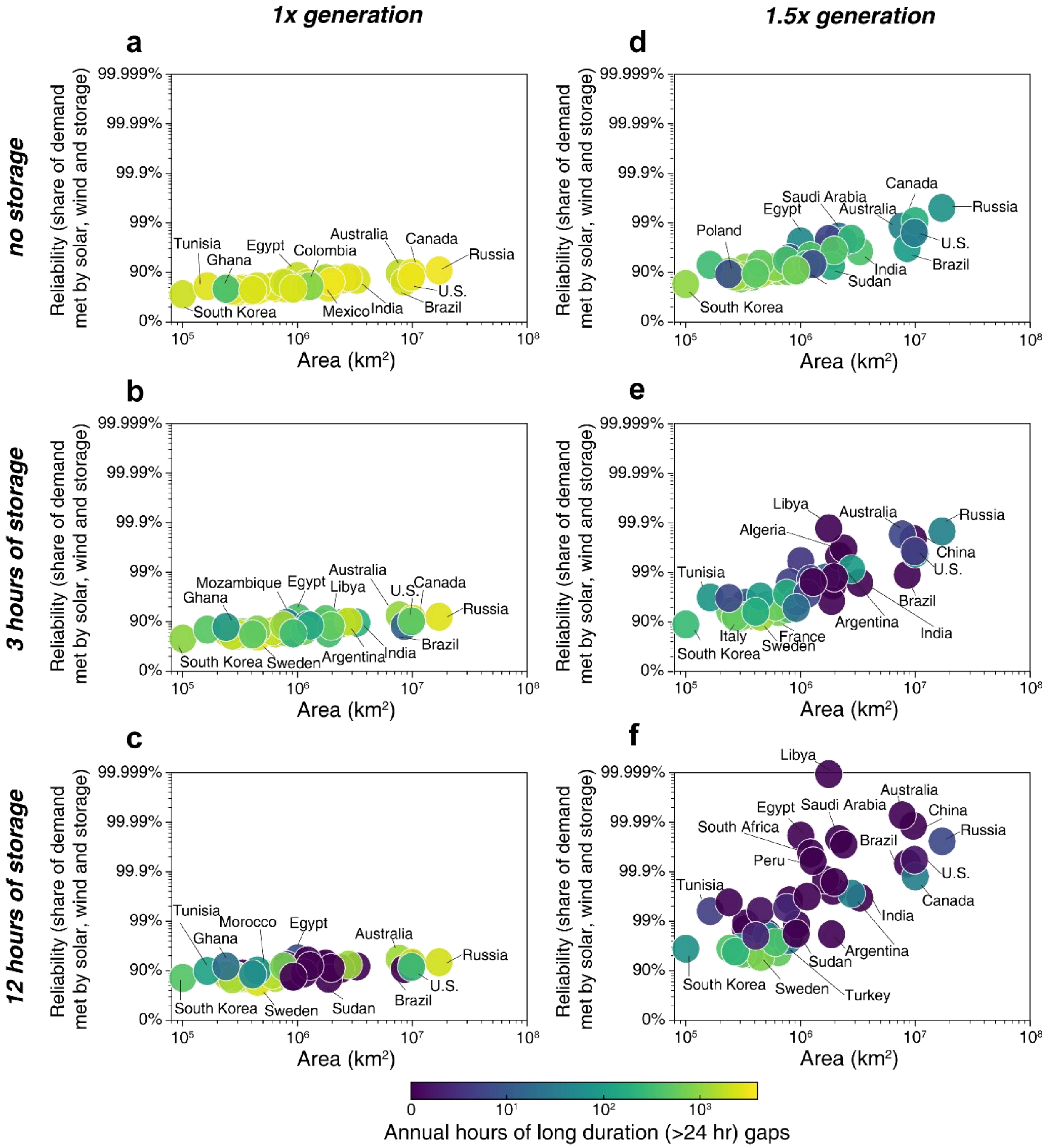

Supplementary Figure 4 | The relationship between the highest reliability of electricity supply system and the national area among 42 major countries. Shading of bubbles represents the annual average hours of long-duration (>24 hours) power supply gaps. Storage and generation quantities are varied in each panel: (a) 1 x generation without storage; (b) 1 x generation with 3 hours of storage; (c) 1 x generation with 12 hours of storage; (d) 1.5 x generation without storage; (e) 1.5 x generation with 3 hours of storage; and (f) 1.5 x generation with 12 hours of storage.

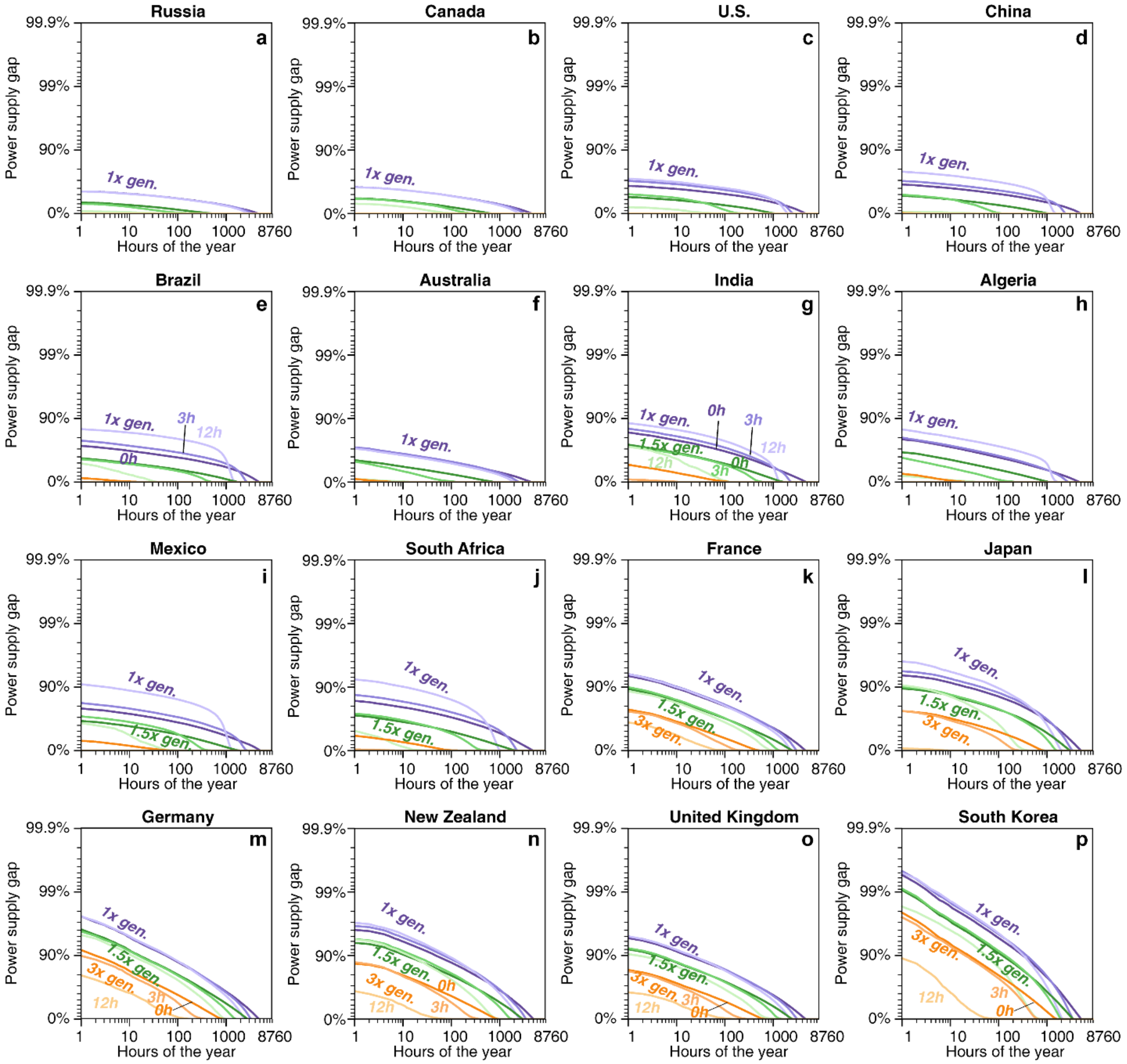

Supplementary Figure 5 | Average power supply gap. Areas under each curve show the share and hours of unmet electricity demand of the most reliable solar-wind systems in selected countries assuming specified storage and generation quantities: (a) Russia; (b) Canada; (c) contiguous U.S.; (d) China; (e) Brazil; (f) Australia; (g) India; (h) Algeria; (i) Mexico; (j) South Africa; (k) France; (l) Japan; (m) Germany; (n) New Zealand; (o) United Kingdom; (p) South Korea. Color of lines represents different generation quantities: 1x generation in purple, 1.5x generation in green, and 3x generation in orange. Shading of lines represents different storage quantities: darkest shading represents without storage, medium shading represents 3 hours of storage, and lightest shading represents 12 hours of storage. Note that the y-axis of power supply gap represents the fraction of unmet demand to the total demand in that hour.

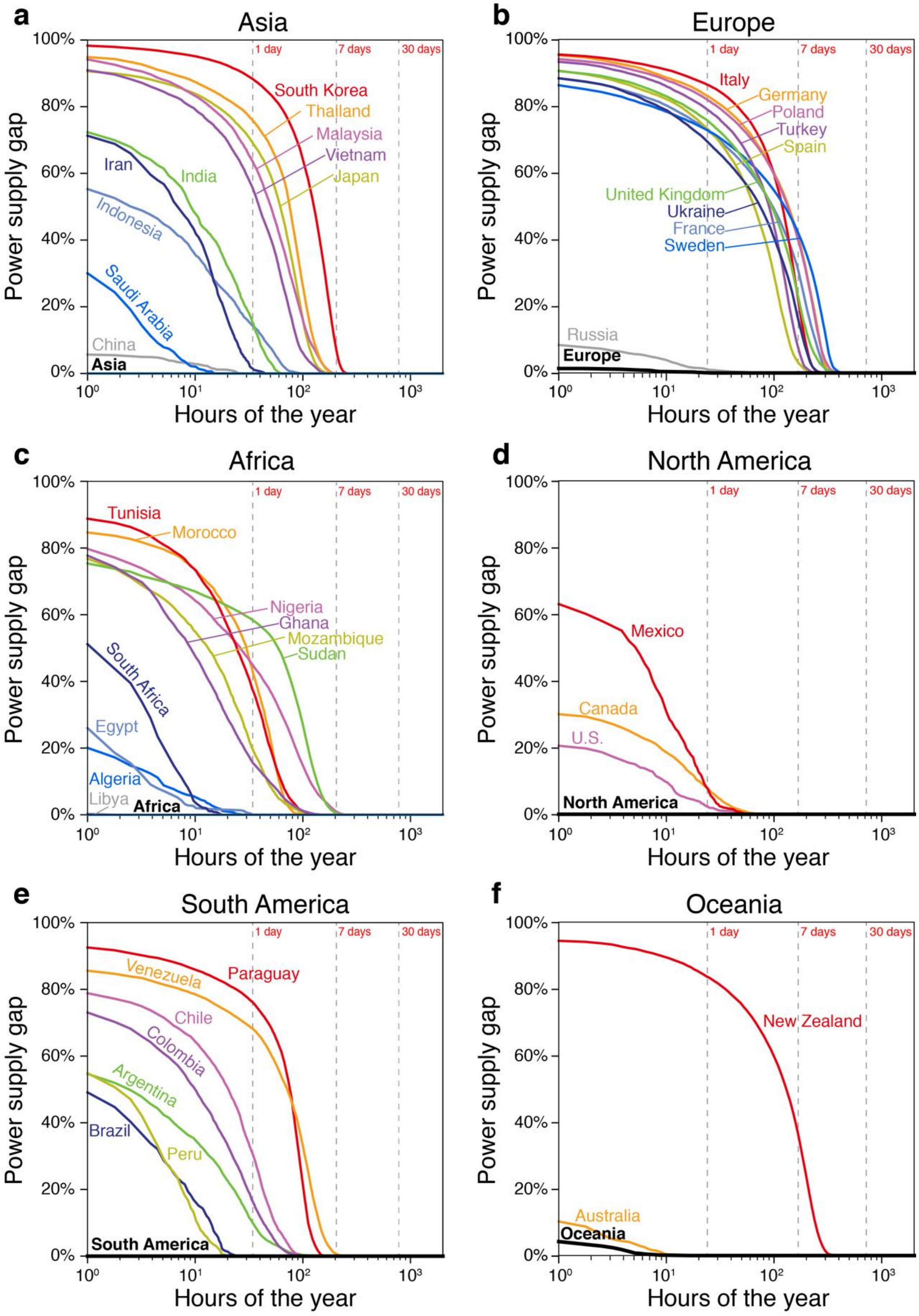

Supplementary Figure 6 | Average power supply gap. The power supply gaps under the most reliable electricity systems for six continents with 12 hours of storage and 50% of capacity margin are shown.

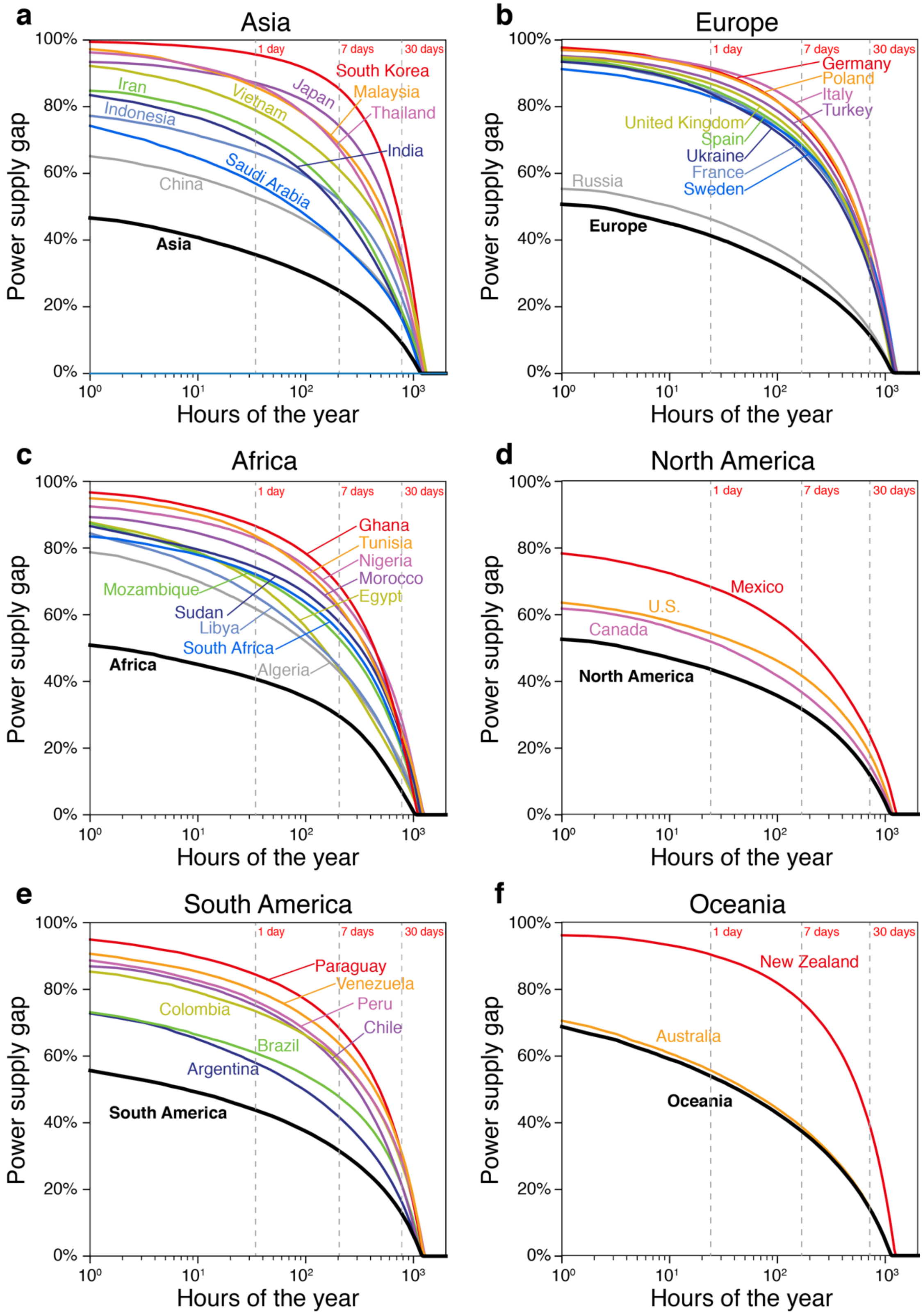

Supplementary Figure 7 | Average power supply gap. The power supply gaps under the most reliable electricity systems for six continents with no storage and excess of capacity margin are shown.

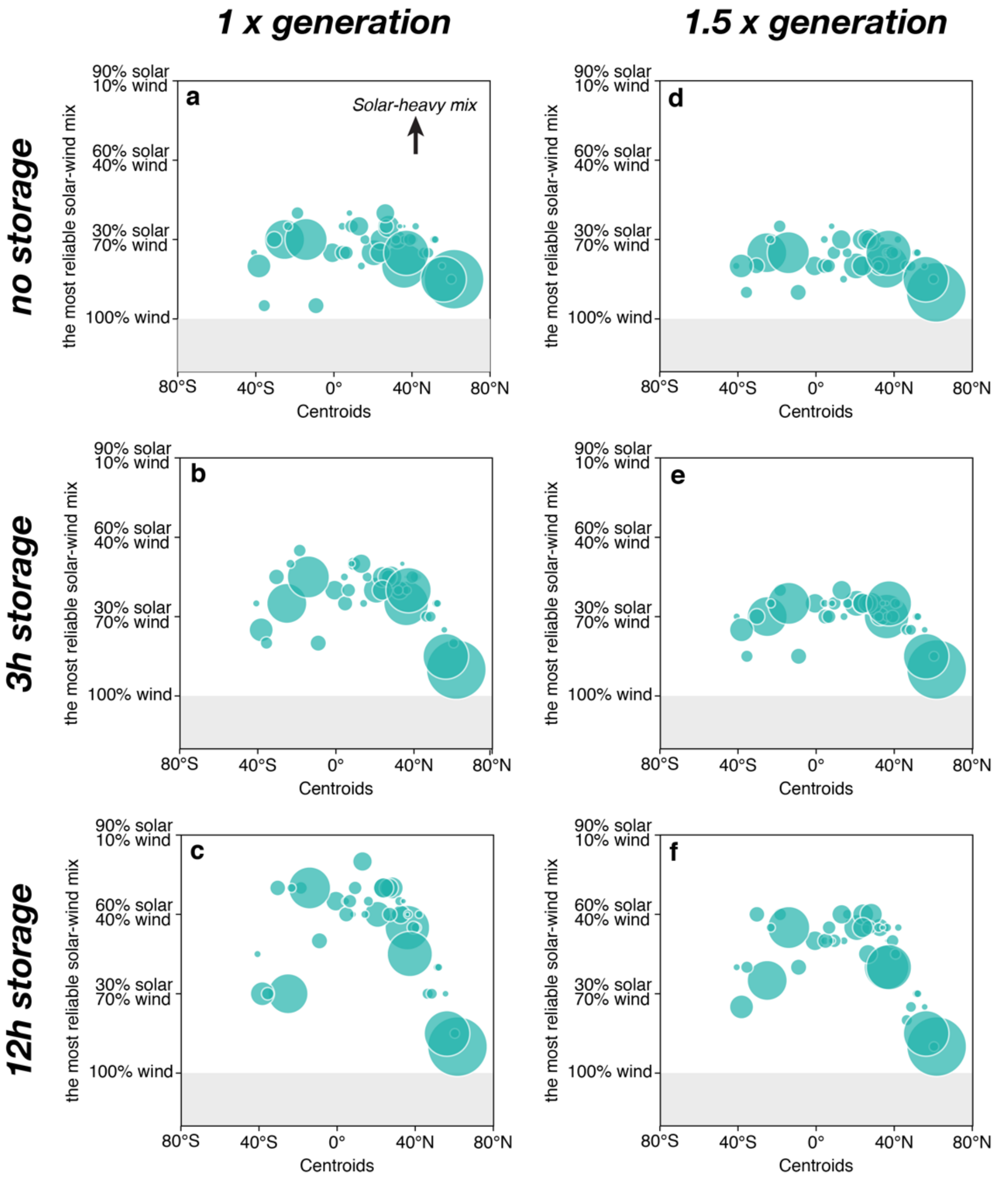

Supplementary Figure 8 | The most reliable solar-wind mix and the latitude of geographical centroids for 42 major countries. Storage and generation quantities are varied in each panel: (a) 1 x generation without storage; (b) 1 x generation with 3 hours of storage; (c) 1 x generation with 12 hours of storage; (d) 1.5 x generation without storage; (e) 1.5 x generation with 3 hours of storage; and (f) 1.5 x generation with 12 hours of storage. The sizes of bubbles represent relative land areas.

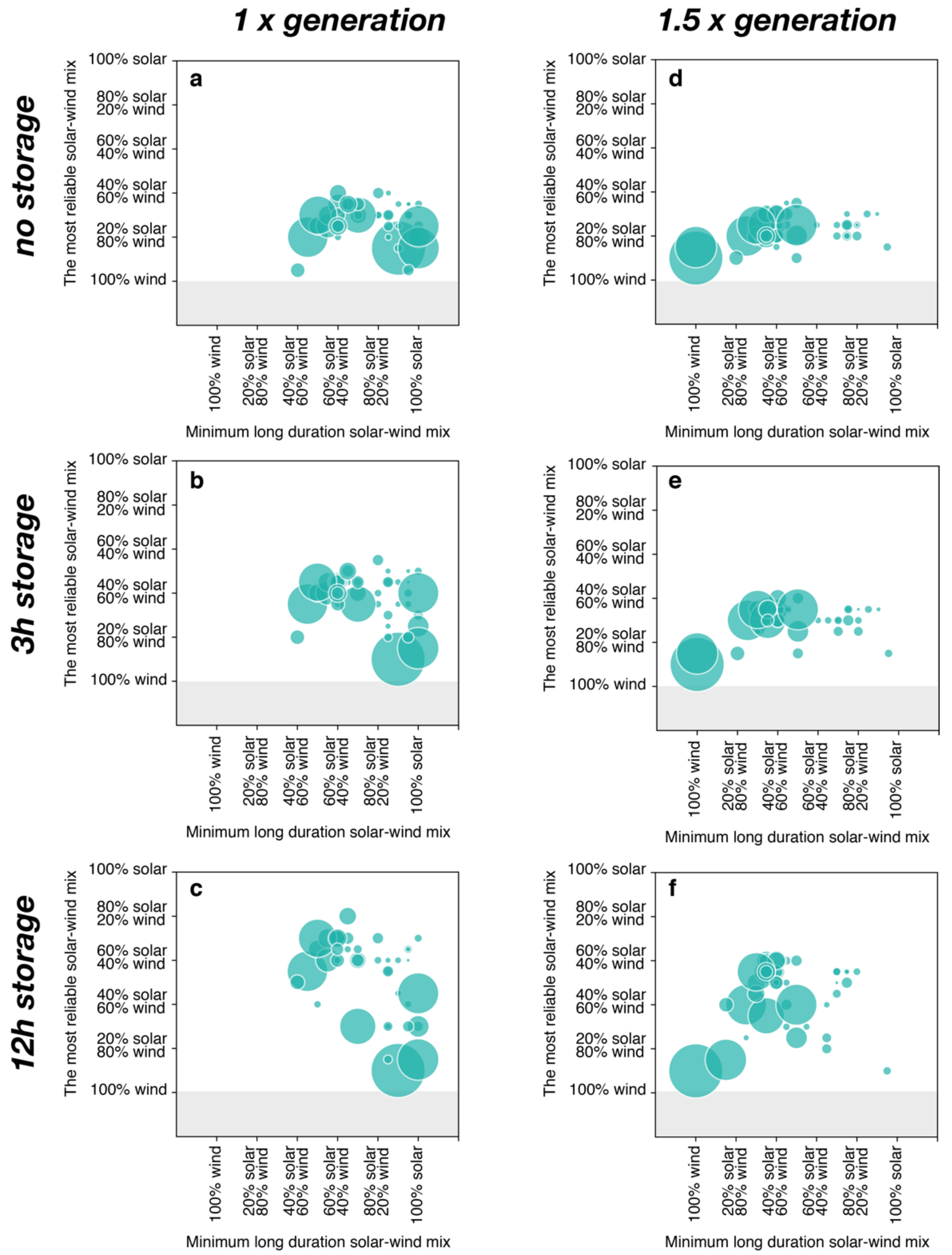

Supplementary Figure 9 | Comparisons between the most reliable and minimum long duration (≥24h) solar-wind mix. Storage and generation quantities are varied in each panel: (a) 1 x generation without storage; (b) 1 x generation with 3 hours of storage; (c) 1 x generation with 12 hours of storage; (d) 1.5 x generation without storage; (e) 1.5 x generation with 3 hours of storage; and (f) 1.5 x generation with 12 hours of storage. The sizes of bubbles represent relative land areas.

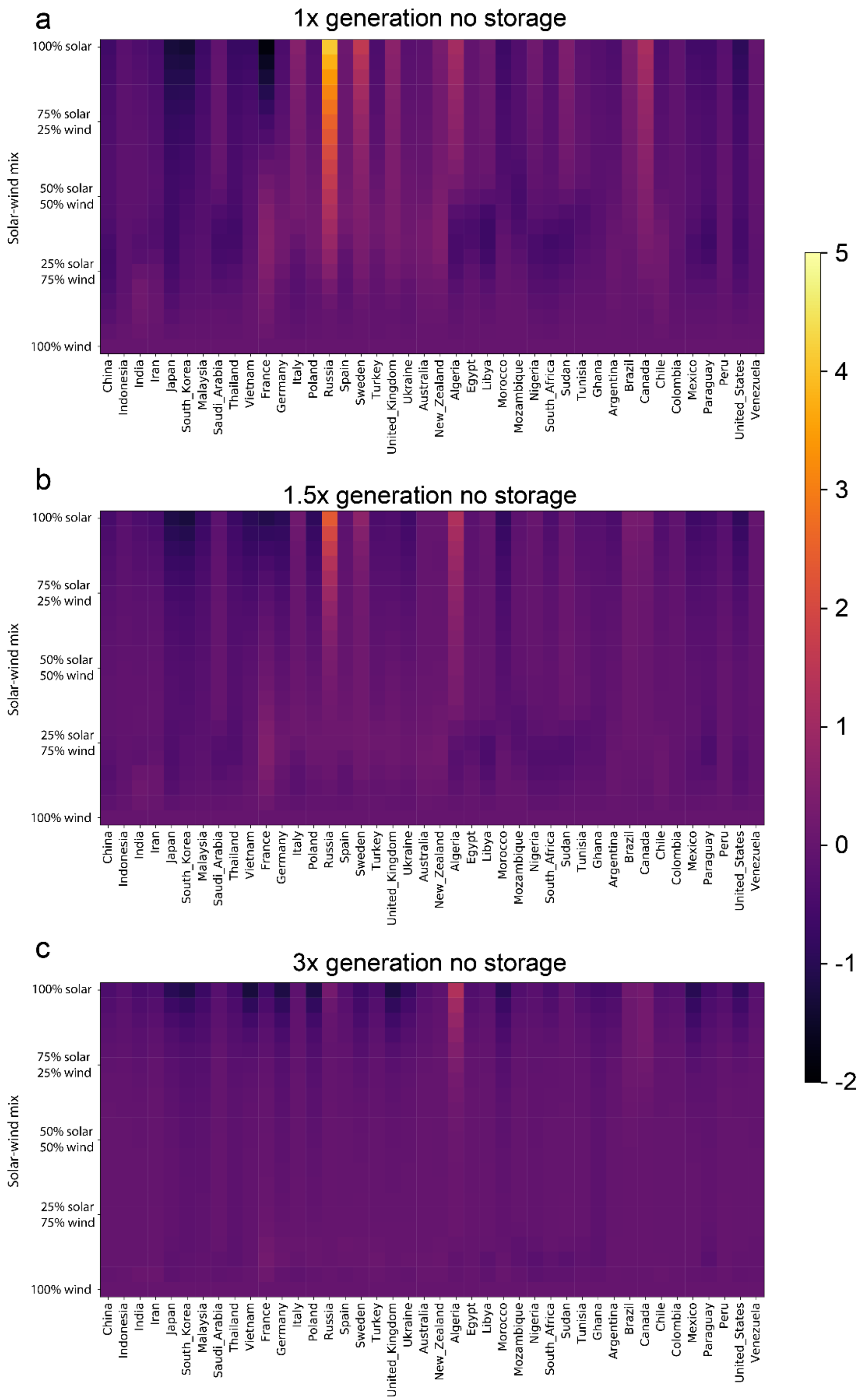

Supplementary Figure 10 | The impacts of solar tracking systems (single-axis and 2-axis) on the system reliability in the 42 major countries. generation quantities are varied in each panel: (a) 1 x generation without storage; (b) 1.5 x generation without storage; (c) 3 x generation without storage. The values mean the reliability change ratio (%) of 2-axis solar tracking system comparing to single axis solar tracking system.

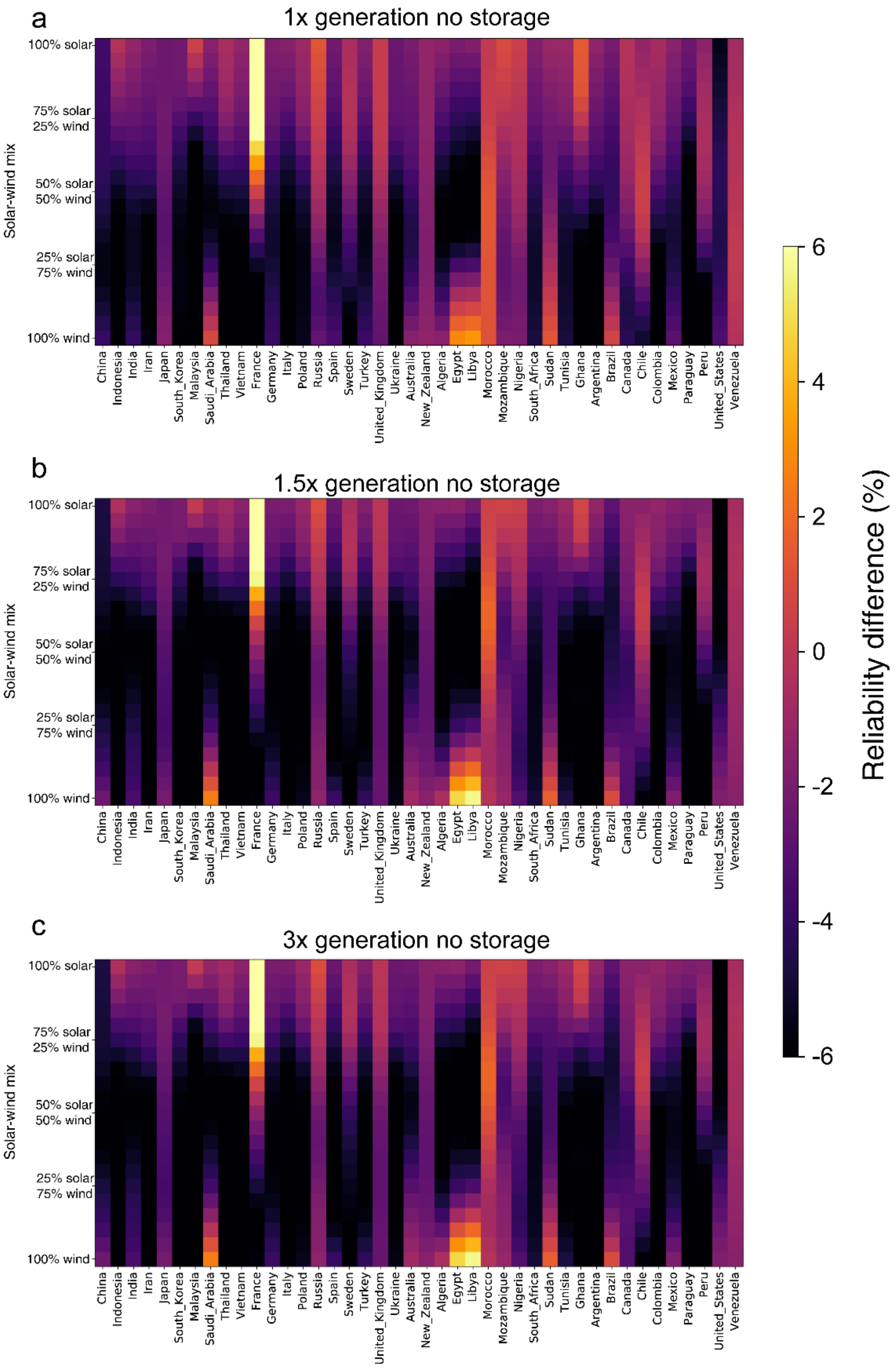

Supplementary Figure 11 | The impacts of different reanalysis data products (MERRA-2 and ERA5) on the system reliability in the 42 major countries. generation quantities are varied in each panel: (a) 1 x generation without storage; (b) 1.5 x generation without storage; (c) 3 x generation without storage. The values mean the reliability change ratio (%) of MERRA-2 comparing to ERA5.

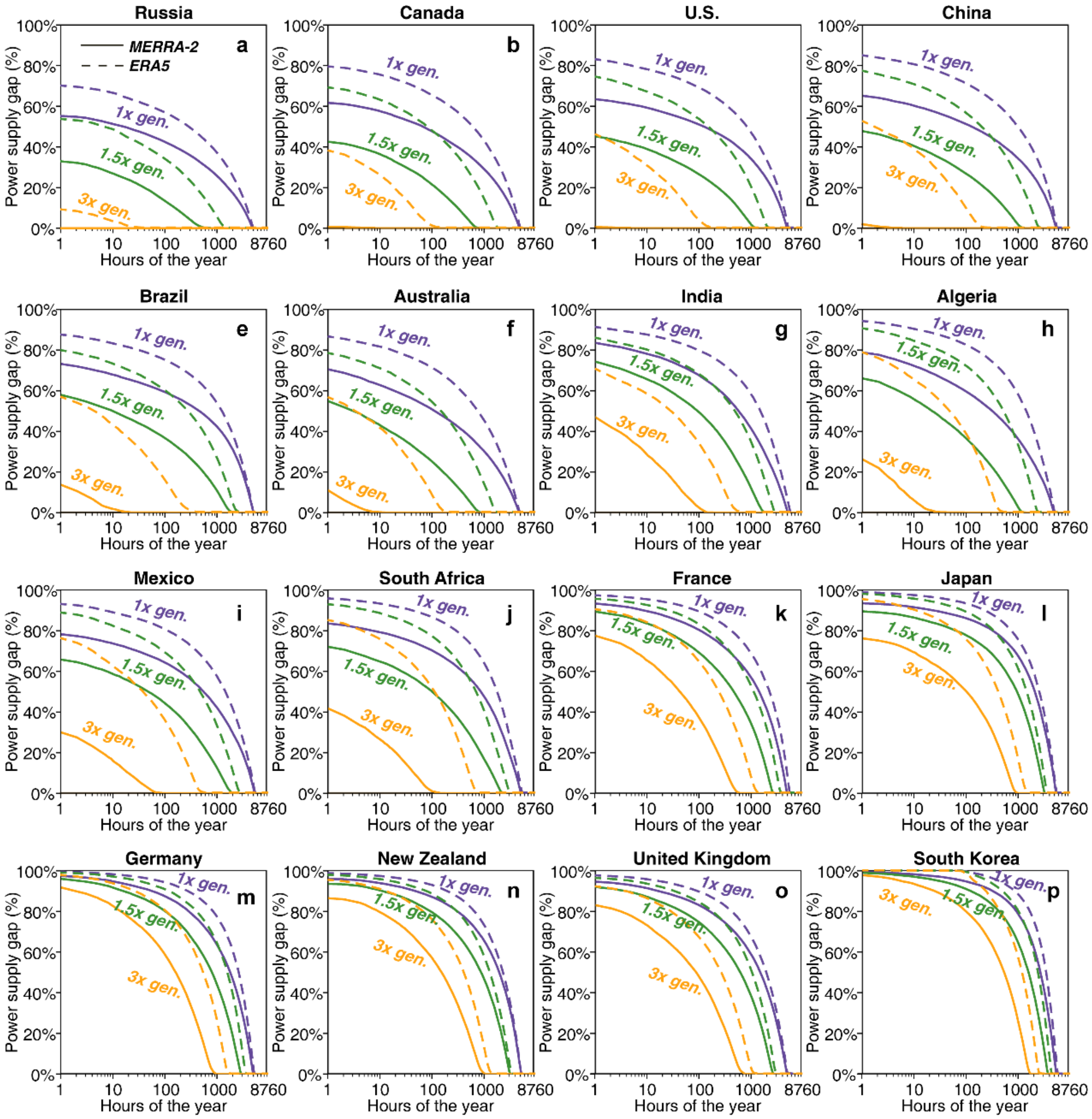

Supplementary Figure 12 | Average power supply gap comparison. The power supply gap comparison of the most reliable electricity system are shown by generation quantity by using reanalysis data MERRA-2 and ERA5: (a) Russia; (b) Canada; (c) contiguous U.S.; (d) China; (e) Brazil; (f) Australia; (g) India; (h) Algeria; (i) Mexico; (j) South Africa; (k) France; (l) Japan; (m) Germany; (n) New Zealand; (o) United Kingdom; (p) South Korea.

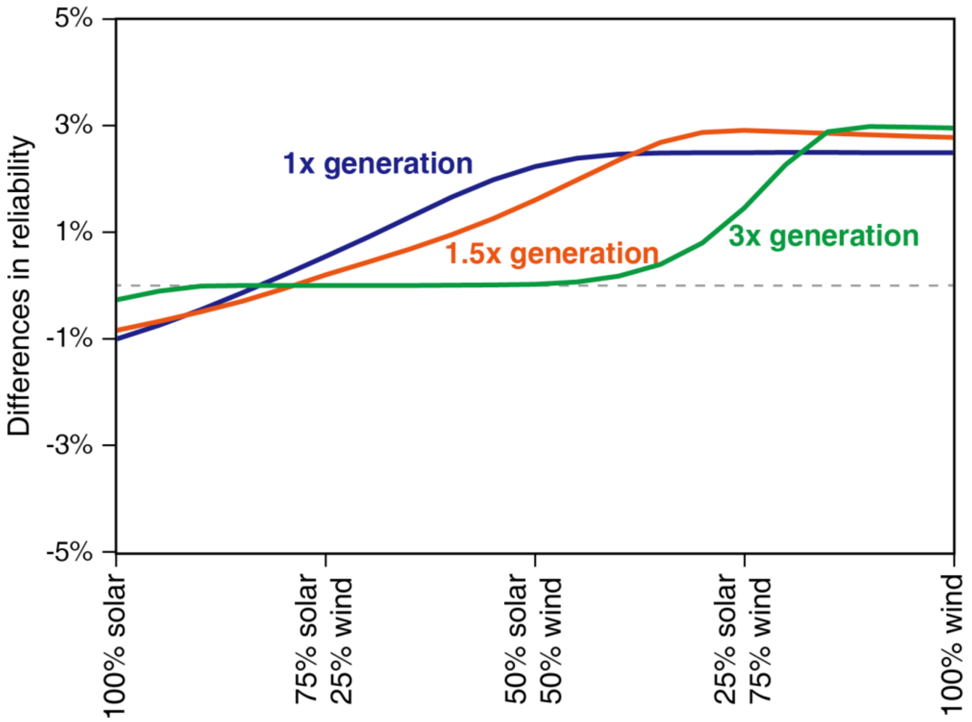

Supplementary Figure 13 | The impacts of future high electrification demand profile on the system reliability in the U.S..

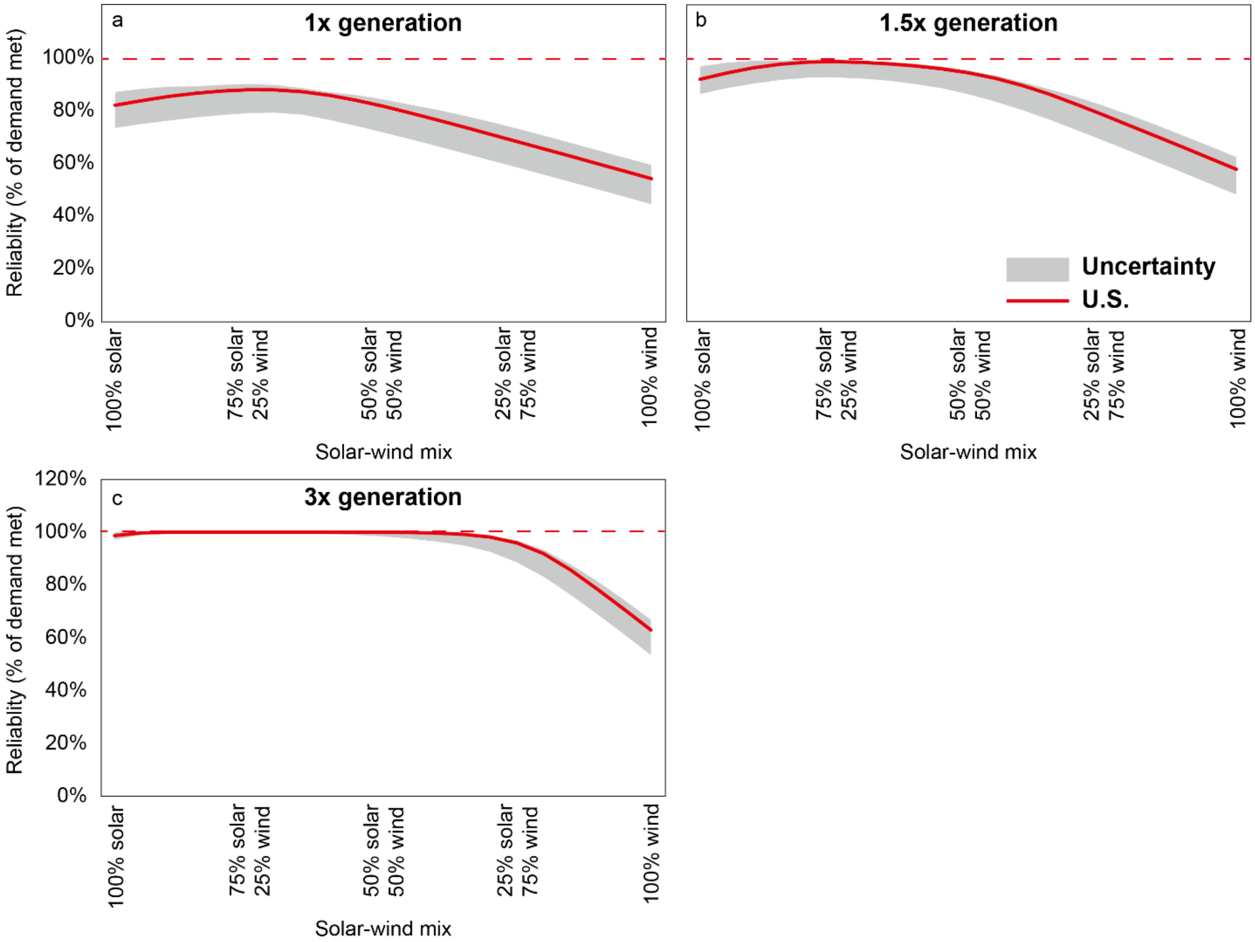

Supplementary Figure 14 | The impacts of various demand characteristics on the system reliability in the U.S..

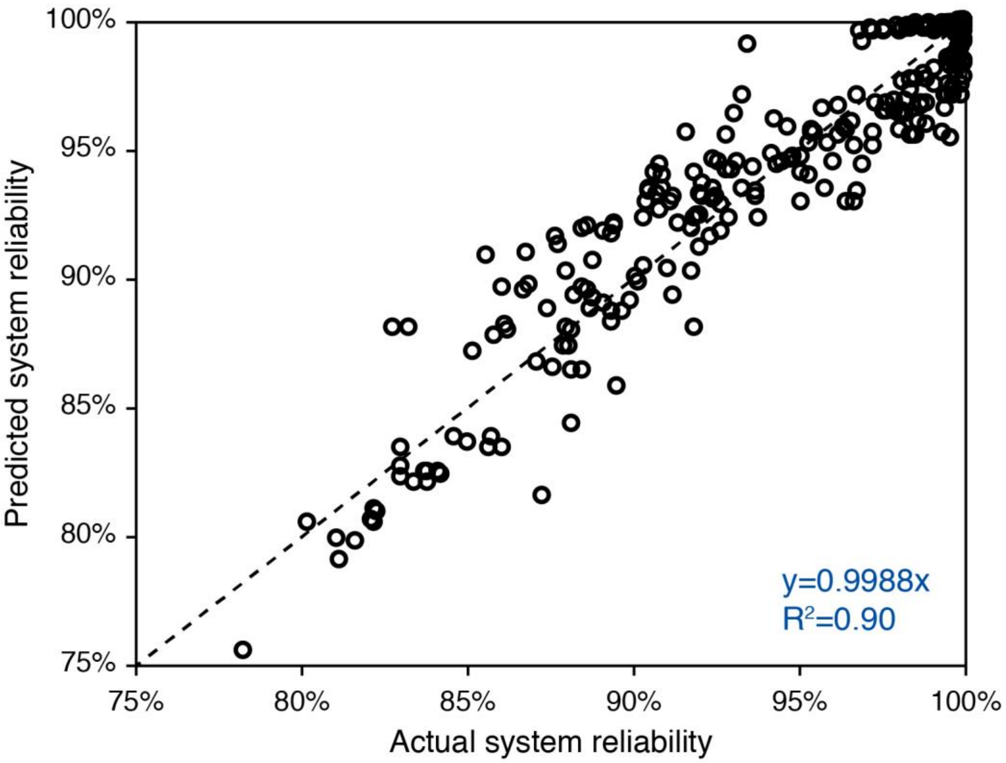

Supplementary Figure 15 | Comparisons between the predicted system reliability and actual system reliability from eq. (S1).
