# Supplementary material for: Geophysical constraints on the reliability of solar and wind power worldwide
Source: Nat Commun. 2021 Oct 22;12:6146. doi: 10.1038/s41467-021-26355-z (PMC8536784; doi:10.1038/s41467-021-26355-z)
Supplement: Supplementary file 3 — Description of Additional Supplementary Files [file 41467_2021_26355_MOESM3_ESM.pdf]

Description of Additional Supplementary Files

File Name: Supplementary Data 1
Description: Summary of collected country (or region)-level electricity demand data.

File Name: Supplementary Data 2
Description: The estimates of Kendall correlation coefficients between solar and wind resources.

File Name: Supplementary Data 3
Description: Summary of land area and system reliability.

File Name: Supplementary Data 4
Description: Classification of multinational regions and continents.

File Name: Supplementary Data 5
Description: Summary of installed capacities by varying the solar and wind resource mix, and energy storage.

File Name: Supplementary Data 6
Description: Average hourly power supply gaps.
